# Lateral Antebrachial Cutaneous Nerve injury induced by phlebotomy

**DOI:** 10.1186/1749-7221-2-6

**Published:** 2007-03-14

**Authors:** S Mansoor Rayegani, Arezoo Azadi

**Affiliations:** 1 Physical medicine & rehabilitation Dept., Shohada medical center, Shaheed Beheshti medical university, Tehran, Iran

## Abstract

**Background:**

Phlebotomy is one of the routine procedures done in medical labs daily.

**Case presentation:**

A 52 yr woman noted shooting pain and dysesthesia over her right side anterolateral aspect of forearm, clinical examination and electrodiagnostic studies showed severe involvement of right side lateral antebrachial cutaneous nerve.

**Conclusion:**

Phlebotomy around lateral aspect of antecubital fossa may cause lateral antebrachial cutaneous nerve injury, electrodiagnostic studies are needed for definite diagnosis.

## Background

Although different venipuncture injuries have been reported with routine phlebotomies, there is little information available on peripheral nerve complications. We present a case of phlebotomy-induced severe injury to the lateral antebrachial cutaneous nerve (LACN), in which the diagnosis was made using nerve conduction study. According to our search and knowledge, the use of electrodiagnostic testing for diagnosis of this type of injury, has only been reported one time for radial [1] nerve and twice for lateral antebrachial cutaneous nerve [2,4].

## Case presentation

**Figure 1:**
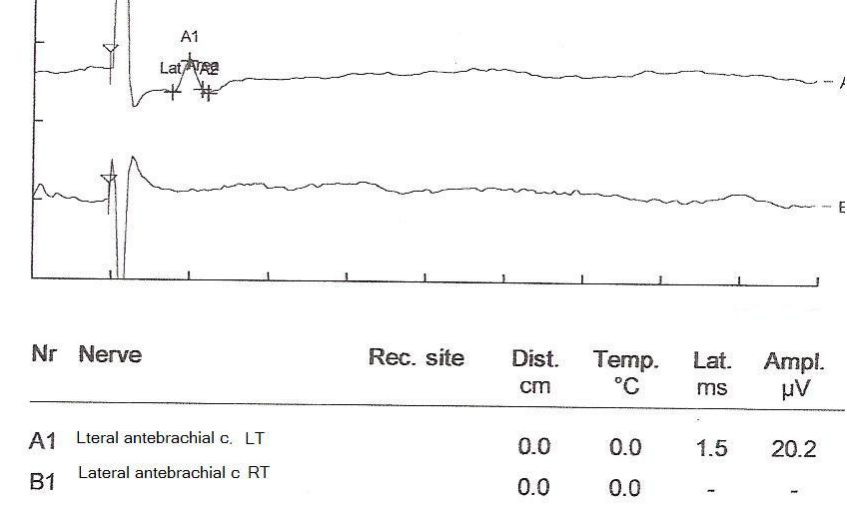
Nerve conduction responses of both sides LACN A1:normal response obtained from left side LACN B1:no response obtained by right side LACN stimulation.

At the time of venipuncture from Right side cephalic vein in the lateral aspect of the antebrachial fossa, a 52 yr right handed woman complains of shooting pain and dysesthesia over the lateral aspect of right forearm. Twenty days after phlebotomy, she was referred for electrodiagnostic study about possible peripheral nerve damage. Physical examination showed normal inspection, range of motion and manual muscle testing of right upper limb, but decreased sensation to light touch and pin prick limited to the anterolateral aspect of right forearm(distribution of the LACN). Electrodiagnostic study was performed on bilateral LACNs using routine technique [3] by Synergy EMG machine. The study revealed absence of sensory nerve action potential from the right LACN, and normal in left side (figure 1). To ensure that the response is truly absent, stimulation current was eventually increased up to 50 mA and stimulation to 0.3 ms, averaging was used and stimulation was systemically performed at various locations across the antebrachial region to ensure that the nerve was not simply missed. To asses for neurapraxic injury, stimulation was similarly performed down in the forearm distal to the suspected injury site, but still no response could be obtained on the right side. All other nerves in Right upper limb were normal in nerve conduction studies. The diagnosis was severe injury to right LACN. The patient has not returned for further evaluation.

## Discussion

The musculocutaneous nerve can be damaged by a number of mechanisms but injury in isolation is rare compared to other peripheral nerves. It may be injured in the axilla as it pierces the corachobrachialis muscle, or more distally where just the sensory branch (LACN) is affected resulting only in an altered sensation. (3) Anterior dislocation of the shoulder can result in axonal damage to the musculocutaneous nerve as well as the axillary nerve. A number of isolated musculocutaneous nerve injury also has been reported secondary to weight lifting, malpositioning during anesthesia and traumatic arm extension [3]. the nerve is also involved in neuralgic amyotrophy. Rarely an anomalous portion of the biceps brachii muscle may injure LACN. The LACN may be injured during antebrachial phlebotomy [3,4].

Phlebotomy related nerve injuries have been reported for both the routine venipuncture and blood donation populations. These have included injury to LACN, Medial antebrachial cutaneous nerve, superficial radial nerve, and dorsal ulnar sensory branch in the hand [5,6]. Incidence rate have not been quoted for routine phlebotomy patients.

A compressive neuropathy after phlebotomy was also reported in a patient who received oral anticoagulants. In contrast, the two studies on the blood donation population did not specify the particular nerves that were injured, but reported incidences of nerve injury in general after blood donation ranges from1/6300 to approximately 1/25000 donation [6,7].

Nerves are susceptible to injury during phlebotomy because they lie on a plane just beneath and in close proximity to the veins, where they are vulnerable to injury during this procedure [8].

It has been suggested that during phlebotomy, the needle should be placed superficially and the medial aspect of the antecubital fossa should be avoided to avoid injuring medial antebrachial cutaneous nerve [6].

However our case suggests that using the lateral aspect of the fossa puts LACN at the risk of injury. The LACN is the distal sensory extension of the musculocutaneous nerve piercing the deep fascia and emerging from underneath the lateral aspect of the biceps tendon at the level of interepicondylar line. LACN is susceptible to injury when venipuncture involves the portion of cephalic vein that lies just lateral to the biceps tendon and crosses LACN.

In general, phlebotomists should consider that multiple attempts at entering a vein could be associated with a high incidence of direct traumatic nerve injury and also secondary compressive hematoma. Minimizing needle movement is also suggested.

## Conclusion

Although venipuncture-related nerve injuries apparently occur infrequently, electromyographers and other related clinicians should be aware of this uncommon but clinically and medico legally important phenomenon. This condition is probably under recognized because the nerve is purely sensory and there is no motor abnormality. Patients should be informed before phlebotomy that excessive swelling after venipuncture or any new neurologic symptoms should be reported early on. To prevent this injury we suggest that during routine antecubital phlebotomy, the area immediately lateral to the biceps tendon and medial to brachioradialis muscle be avoided. If phlebotomy is to be performed in this location an attempt should be made to do it as superficial as possible [4].

Electrodiagnostic studies should be routinely used in patients complaining of neurologic symptoms at least 10 days after venipuncture to diagnose the location and severity of the injury. More common use of electrodiagnostic studies in all patients with sensory complaints after phlebotomy may ultimately help to establish injury rates with greater precision, although further research would be needed to determine how such testing would alter patient treatment, prognosis or costs.

## References

[B1] Edwards WC Fleming LL Radial nerve palsy at the elbow following venipuncture-case report J Hand Surgery [Am] 1981 2 486 9 10.1016/s0363-5023(81)80105-0 6268698

[B2] Stitik TP Foye PM Nadler SF Bruchman GO Phlebotomy related lateral antebrachial cutaneous nerve injury-case report Am J Phy Med Rehabil 2001 2 3 230 4 10.1097/00002060-200103000-00016 11237278

[B3] Dumitru Daneil Electrodiagnostic medicine 2002 second HANLEY & BELFUS

[B4] Sander HW Conigliari M Masdeu JC Antecubital phlebotomy complicated by lateral antebrachial cutaneous neuropathy N Engl J Med 1998 2 2024 10.1056/NEJM199812313392714 9882205

[B5] Yuan RT Cohen MJ lateral antebrachial cutaneous nerve injury as complication of phlebotomy Plast Reconstr surg 1985 2 299 300 10.1097/00006534-198508000-00024 4023102

[B6] Berry PR Walls WE Venipuncture nerve injuries The Lancet 1977 2 1236 7 10.1016/S0140-6736(77)92442-4 68334

[B7] Newman BH Waxman DA Blood donation-related neurologic needle injury: evaluation of 2 years worth of data from a large blood center Transformation 1996 2 123 5 10.1046/j.1537-2995.1996.36396182137.x 8604504

[B8] Horowitz SH peripheral nerve injury and causalgia secondary to routine venipuncture Neurology 1994 2 962 4 8190306 10.1212/wnl.44.5.962

